# Consistency of the anesthesia consciousness index versus the bispectral index during laparoscopic gastrointestinal surgery with sevoflurane anesthesia: A prospective multi-center randomized controlled clinical study

**DOI:** 10.3389/fnagi.2023.1084462

**Published:** 2023-03-09

**Authors:** Jian Zhan, Feng Chen, Zhuoxi Wu, Zhenxin Duan, Qiangting Deng, Jun Zeng, Lihong Hou, Jun Zhang, Yongyu Si, Kexuan Liu, Mingjun Wang, Hong Li

**Affiliations:** ^1^Department of Anesthesiology, Second Affiliated Hospital of Army Medical University, Chongqing, China; ^2^Department of Anesthesiology, Affiliated Hospital of Southwest Medical University, Luzhou, Sichuan, China; ^3^Editorial Office of Journal of Army Medical University, Army Medical University, Chongqing, China; ^4^Department of Anesthesiology, West China Hospital of Sichuan University, Chengdu, Sichuan, China; ^5^Department of Anesthesiology, Xijing Hospital of Air Force Military Medical University, Xi’an, Shanxi, China; ^6^Department of Anesthesiology, Fudan University Shanghai Cancer Center, Shanghai, China; ^7^Department of Anesthesiology, Second Affiliated Hospital of Kunming Medical University, Kunming, China; ^8^Department of Anesthesiology, Nanfang Hospital of Southern Medical University, Guangzhou, China; ^9^Department of Anesthesiology, Chinese People’s Liberation Army General Hospital, Beijing, China

**Keywords:** depth of anesthesia, anesthesia consciousness index, bispectral index, sevoflurane, consistency

## Abstract

**Background:**

This study aimed to compare the consistency of anesthesia consciousness index (Ai) with that of bispectral index (BIS) in monitoring the depth of anesthesia (DOA) during sevoflurane anesthesia, to reveal the optimal cutoff values in different states of consciousness, and explore the stability of DOA monitoring during intraoperative injurious stimulation.

**Methods:**

We enrolled 145 patients (97 men and 48 women) from 10 medical centers. General anesthesia was induced using intravenous anesthetics and maintained with sevoflurane. Ai and BIS values were recorded.

**Results:**

The mean difference between the Ai and BIS was-0.1747 (95% confidence interval, −0.6660 to 0.3166; *p* = 0.4857). The regression equation of Ai and BIS from the Deming regression analysis was *y* = 5.6387 + 0.9067*x* (*y* is BIS, *x* is Ai), and the slope and intercept were statistically significant. Meanwhile, the receiver operating characteristic curve analysis of anesthesia-induced unconsciousness, loss of consciousness, and recovery of consciousness revealed that the accuracy of Ai and BIS were similar. In addition, the optimal cutoff values of the different states of consciousness were not sensitive to age, and both Ai and BIS had no correlation with hemodynamics.

**Conclusion:**

We conclude that Ai and BIS show no systematic deviation in readings with high consistency, similar accuracy, and good stability; these insights provide more data for clinical application.

## Introduction

To prevent intraoperative awareness of patients under general anesthesia, an accurate monitoring of the depth of anesthesia (DOA) is crucial, thereby maintaining an appropriate DOA, reducing the number of anesthetics and anesthesia-related complications, and accelerating postoperative recovery (Myles et al., 2004; Radtke et al., 2013; Lewis et al., 2019). Electroencephalogram (EEG) signals reflect the effects of general anesthetics on the central nervous system and accurately monitor changes in the patient’s state of consciousness (Brown et al., 2010, 2011). Various anesthesia depth indices have been developed based on EEG signals. The bispectral index (BIS) is the first anesthesia depth index certified by the United States Food and Drug Administration and is thus often used as a reference standard for evaluating the accuracy of anesthesia depth monitoring (Myles et al., 2004; Powers et al., 2005; Brown et al., 2010, 2011; Radtke et al., 2013; Lewis et al., 2019). However, advanced age, the performance of response to stress diverges, neurovascular diseases, and intracranial pathology are some potential interference factors, and many anesthetic drugs produce characteristic effects on the EEG. Further research revealed important limitations of this technology (Katoh et al., 1999; Bowdle, 2006; Bennett et al., 2008; Leece et al., 2008); therefore, an effective DOA monitor is needed to guide anesthetic titration for optimal clinical care and minimal hospitalization costs.

The anesthesia consciousness index (Ai) is a new quantitative index that assesses brain waves from an awake state to the deepest state of anesthesia based on sample entropy (Liu et al., 2015). Entropy measures the randomness or irregularity of the signals. Increasing anesthesia depth correlates with decreased randomness, with the EEG signals displaying more regularity, implying a more stable and predictable system (Fahy and Chau, 2018). It has been reported that BIS cannot predict the exact moment of consciousness recovery (Johansen, 2006), whereas Ai can better reflect the altered state of consciousness (Shalbaf et al., 2012; Jiang et al., 2015). Therefore, although it has been established that the accuracy of Ai in monitoring propofol-induced depth is not significantly different from that of BIS (Fu et al., 2019), Ai may perform better than BIS in monitoring the DOA.

However, an accurate parameter for anesthesia depth monitoring under inhalational anesthetics has not been reported, and whether it is affected by intraoperative hemodynamics remains unclear. Therefore, this study aimed to evaluate the consistency and accuracy of Ai and BIS in monitoring the DOA during sevoflurane administration, to explore the stability of these indices in monitoring the DOA during intraoperative noxious stimulation, and to reveal the optimal cutoff values of different consciousness levels to provide a data reference for clinical application.

## Materials and methods

### Participants

This study was registered in the China Clinical Trial Registration Center (ChiCTR2000034839) and approved by the Medical Ethics Committee of the Second Affiliated Hospital of Army Medical University (2020-Research No. 062–01). Informed consents were signed by patients or their guardians. We enrolled 152 patients aged 18–75 years who underwent elective laparoscopic gastrointestinal surgery under general anesthesia (American Standards Association state I or II, body mass index 18.5–24.9 kg/m^2^). No patient had a history of mental or neurological disease, sedative or analgesic drug therapy or abuse, or contraindications for or allergies to sedative or analgesic drugs; language or hearing impairment; or serious heart, lung, kidney, or liver dysfunction.

All patients were randomly assigned to the Second Affiliated Hospital of Army Medical University, West China Hospital of Sichuan University, Nanfang Hospital of Southern Medical University, Chinese People’s Liberation Army General Hospital, the Second Affiliated Hospital of Kunming Medical University, Xijing Hospital of Air Force Military Medical University, Fudan University Shanghai Cancer Center, the First Hospital of Shanxi Medical University, the Fourth Hospital of Hebei Medical University, and Shandong Yantai Yuhuangding Hospital.

### Experimental procedures

The side of the forehead was randomized, the EEG electrode strips for Ai (Conview YY-106, Zhejiang Puke Medical Technology Co., Ltd.) were positioned, and the BIS (VISTA™ monitoring system, Covidien LLC, One Upland Road Norwood, MA 02062, USA) electrodes were placed on the opposite side. Moreover, non-invasive blood pressure, pulse oxygen saturation, electrocardiogram, heart rate, and end-tidal carbon dioxide partial pressure were monitored. The peripheral veins of the upper extremities were opened. Oxygen was provided using a mask without premedication.

Conscious patients were instructed to remain calm with their eyes closed (but not to fall asleep) (Nieuwenhuijs et al., 2002)and to keep their facial muscles completely relaxed(Bruhn et al., 2000). Recordings were started after verifying a sustained low electromyography activity and a signal quality index above 95%. During the induction of anesthesia, patients were instructed to open their eyes, and continuous verbal communication with the anesthetists was maintained. Concurrently, the patient’s state of consciousness was assessed. Loss of consciousness (LOC) was defined as the absence of a response to calling commands (i.e., no response to the “name, name, open eyes” command) (Russell, 2006). Recovery of consciousness (ROC) was defined as a response to the name command (i.e., a response to the “name, name, open eyes” command [eyes open]) (Russell, 2006).

Intravenous injection of propofol (1.5–2.5 mg/kg), followed by sufentanil (0.3–0.5 μg/kg) and cisatracurium (0.15–0.2 mg/kg) were administered after LOC. Anesthesia was maintained with sevoflurane to maintain the DOA at a BIS of 40–60, with further intermittent or continuous administration of sufentanil, remifentanil, and cisatracurium. No other sedatives or analgesics were administered during the surgery. Blood pressure was maintained within 20% of the baseline blood pressure (baseline blood pressure was calculated by taking the average of three non-invasive blood pressure measurements before anesthesia induction) (Bijker et al., 2007; Monk et al., 2015). The end-tidal carbon dioxide partial pressure was 35–45 mmHg and the core temperature was >36°C. Thirty minutes before the end of the surgery, sufentanil (0.1 μg/kg) was intravenously injected as the first dose of postoperative analgesia, and additional muscle relaxants were stopped. Sevoflurane was stopped at the end of the surgery. When awakening from anesthesia, the patient’s state of consciousness was assessed every minute. Tracheal extubation was performed after the patient awakened from anesthesia and met the indications for extubation.

### Data collection

Ai and BIS values were recorded at rest with eyes closed (baseline data) and at the start of induction, LOC, tracheal intubation, surgical skin incision, establishment of pneumoperitoneum, peritoneal irrigation and removal of the endoscope, ROC, and tracheal extubation. Postoperative follow-up was performed on the first and seventh days after the surgery. The researchers followed up the patients and evaluated the occurrence of intraoperative awareness using a modified Brice questionnaire (Mashour et al., 2013).

### Statistical analysis

SPSS 26.0 and MedCalc 15.2 statistical software were used for analysis. Count data and grade data were expressed as frequencies and composition ratios. Continuous variable data are presented as the mean ± standard deviation ( $\bar{x}$ ± s). Bland–Altman consistency analysis and Deming regression analysis were used to analyze the consistency of Ai and BIS. The receiver operating characteristic curve was used to analyze the accuracy and optimal cutoff values of the two indices in judging the state of consciousness. Spearman’s rank correlation analysis was used to analyze the correlation between the two indices and hemodynamics for reflecting the stability. Differences were considered statistically significant at *p* < 0.05.

Bland–Altman consistency analysis of Ai and BIS was the main observation index used to determine the sample size of this study. The Bland–Altman agreement test suggested a sample size of over 100 (Fu et al., 2019). An α of 0.05 and a power (1−*β*) of 0.9 was set for this study. Based on pre-experimental data, the mean of the difference between Ai and BIS was-2.93, and the standard deviation of the difference was 9.28. The difference in various anesthesia depth index values is considered statistically significant if the difference is greater than or equal to 10 (Aho et al., 2015). Based on the Bland–Altman consistency sample size calculation formula, the requisite sample size was 121 cases. Setting a dropout rate of 20%, the total sample size was set at 152 cases.

## Results

### Demographic

Among the 152 patients, seven were excluded, including five whose procedure was converted to laparotomy and two who were lost to follow-up. The sex, age, height, weight, body mass index, American Standards Association classification, BIS electrode sticking position, operation time, and anesthesia time of the participants are shown in Table 1. No intraoperative awareness was observed in any of the subjects.

**Table 1:** Characteristics of patients at baseline.

	*N* (%) or $\bar{x}$ ±s
Men (%)	97(66.9%)
Women (%)	48(33.1%)
Age (years)	56.48 ± 10.38
Height (cm)	164.70 ± 7.60
Weight (kg)	61.86 ± 13.10
BMI (kg/m^2^)	22.71 ± 4.11
ASA status (%)	
I	11(7.6%)
II	134(92.4%)
BIS electrode sticking position	
Left forehead (%)	72(49.7%)
Right forehead (%)	73(50.3%)
Operation time (min)	198.99 ± 89.37
Anesthesia time (min)	225.89 ± 91.01

### Consistency comparison of Ai and BIS

**Figure 1:**
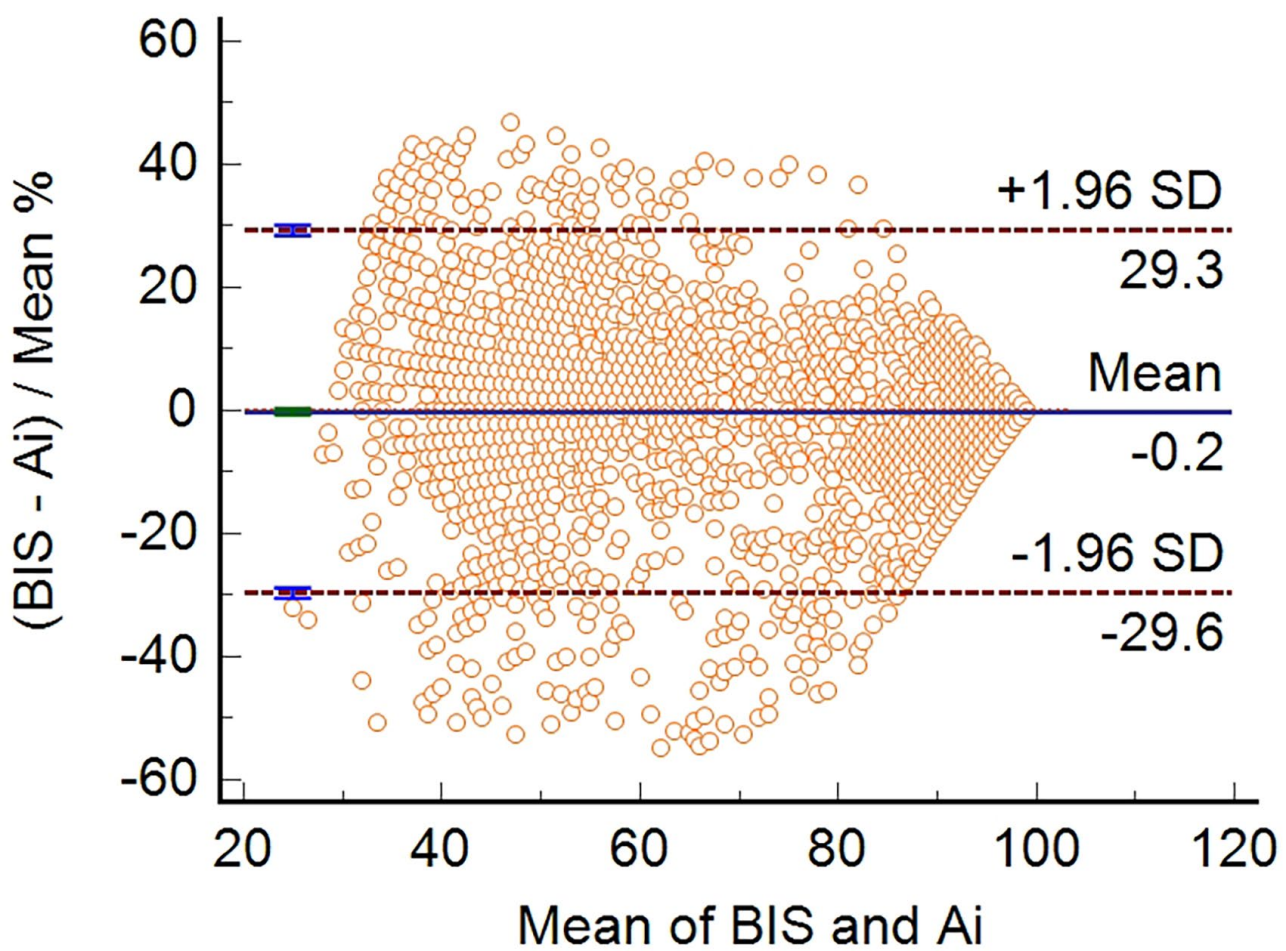
Bland–Altman consistency analysis between Ai and BIS. The bias (mean difference) between the Ai and BIS was-0.1747 (95% CI, −0.6660 to 0.3166) (*n* = 3,600).

Bland–Altman consistency analysis was used to compare the differences between Ai and BIS, and the results showed that the mean difference between the two indices was-0.1747 (95% confidence interval, −0.6660 to 0.3166), *p* = 0.4857. The ±1.96 standard deviation range of the two indices was (−29.6 to 29.3%). Overall, the difference between Ai and BIS scores was not statistically significant (Figure 1).

**Figure 2:**
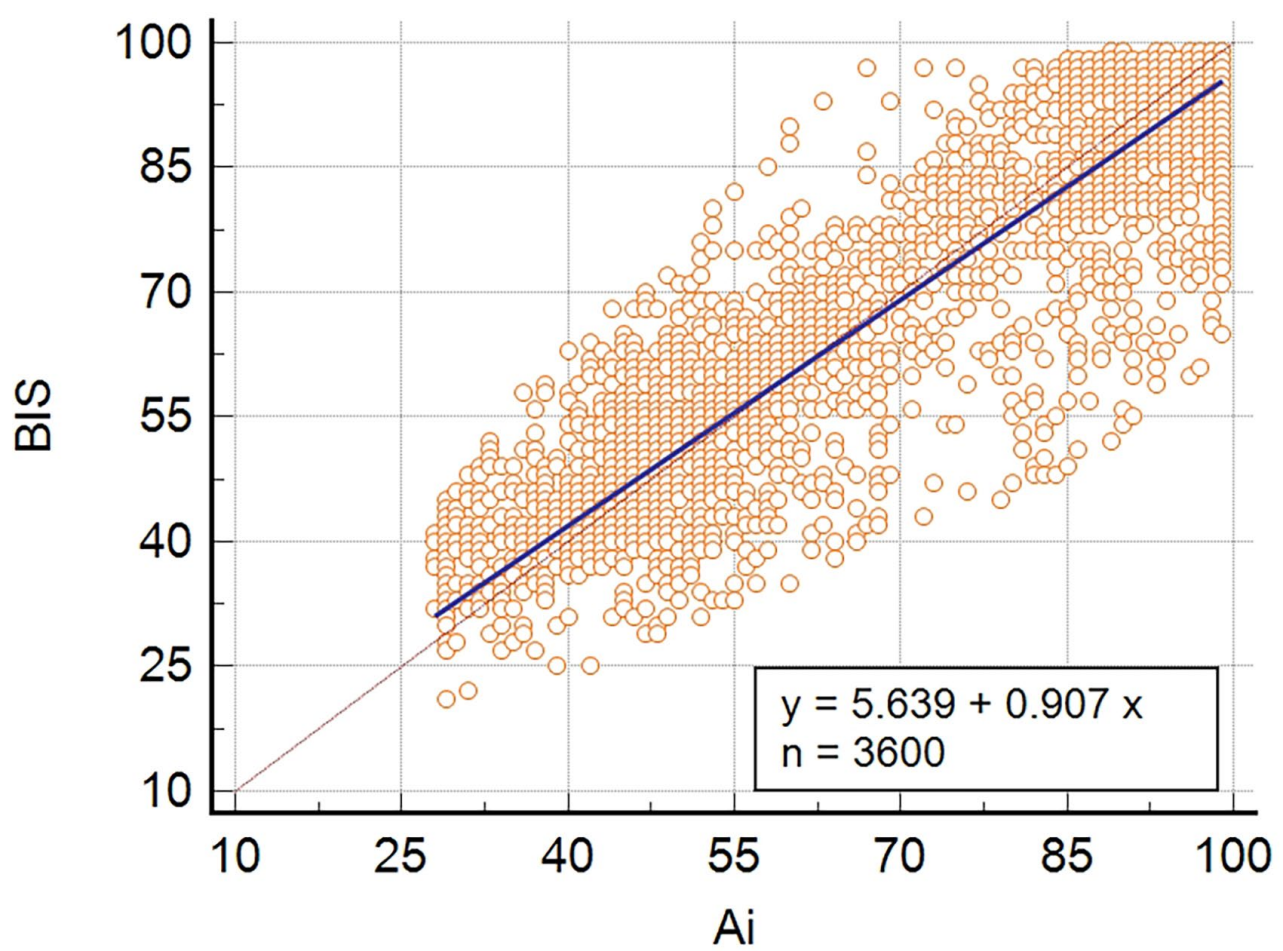
Deming regression analysis of Ai and BIS. The valve of BIS ranged from 21 to 98, and the value of Ai ranged from 29 to 99.

**Figure 3:**
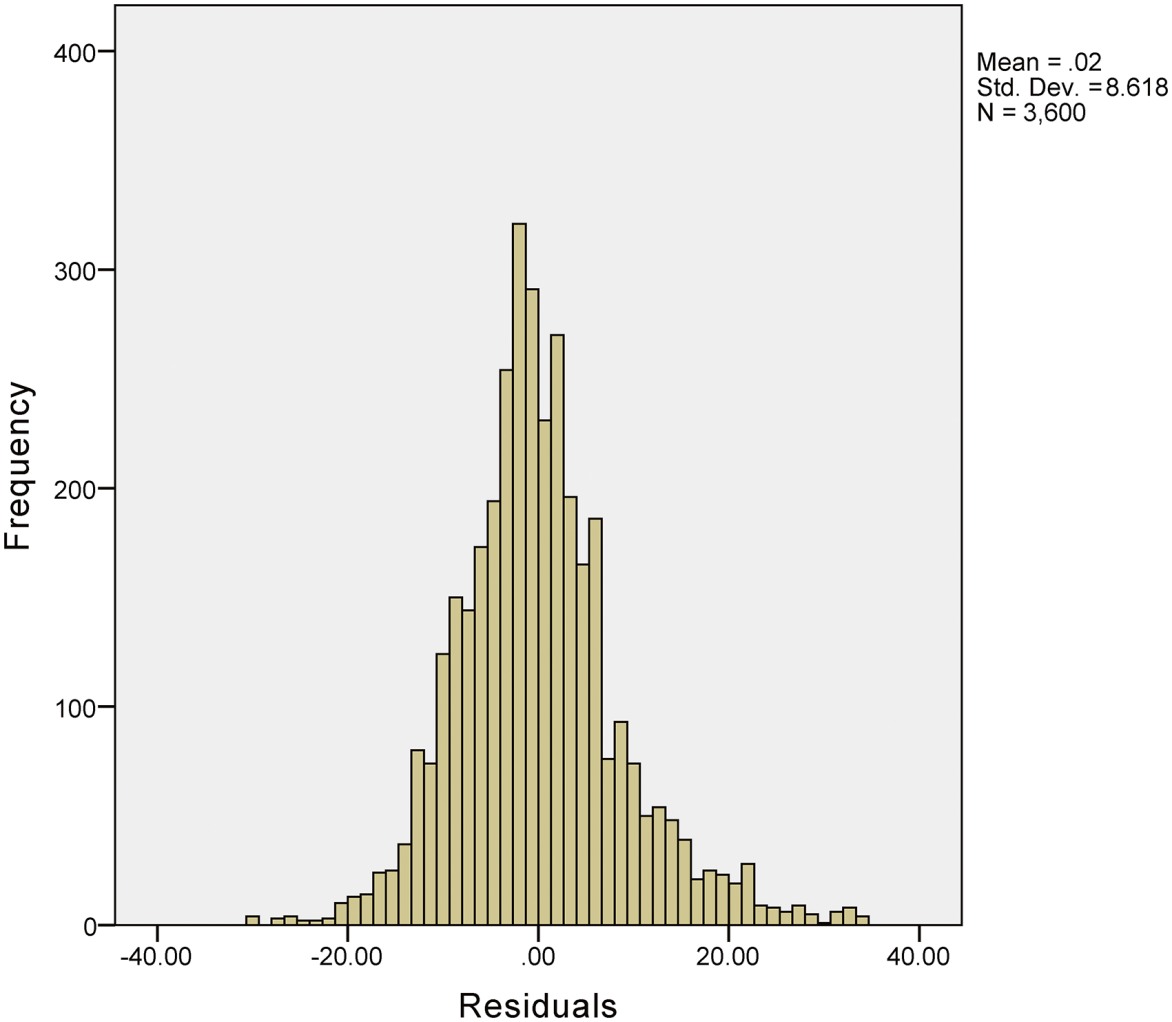
Deming regression-predicted residual values distribution.

Deming regression analysis was used to compare the consistency between Ai and BIS. The results show that the Deming regression equation y = 5.6387 + 0.9067x (y is BIS, x is Ai), the slope, and intercept are statistically significant (Figure 2). Moreover, the residuals were calculated according to the predicted values of the Deming regression equation, and the distribution of the residuals obeyed the normal distribution, with zero as the mean and 8.6 as the standard deviation (Figure 3).

### Accuracy comparison in judging the anesthesia-induced unconsciousness, LOC, and ROC between Ai and BIS

**Figure 4:**
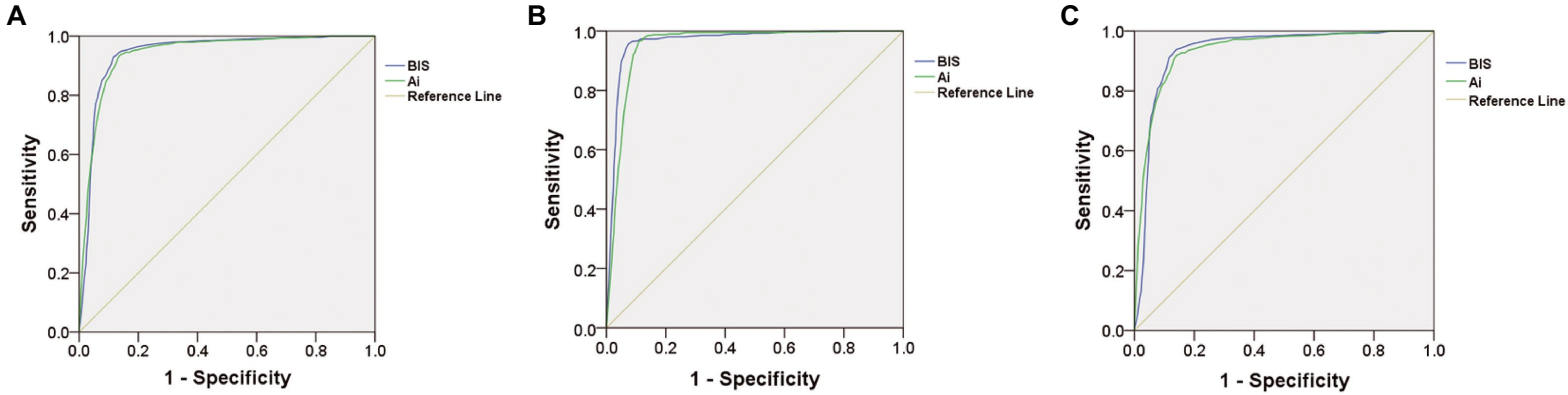
Comparison of receiver operating characteristic curve showing the accuracy in judging the anesthesia-induced unconsciousness **(A)**, LOC **(B)**, or ROC **(C)** between Ai and BIS.

**Figure 5:**
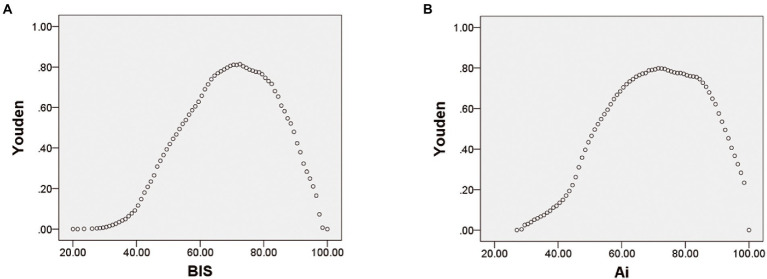
BIS with the Youden index **(A)** and Ai with the Youden index **(B)**.

Receiver operating characteristic curve analysis was used to compare the accuracy of Ai and BIS in judging anesthesia-induced unconsciousness (from LOC to ROC). The results showed that the area under the curve (AUC) of BIS in distinguishing anesthesia-induced unconsciousness was 0.943, and the AUC of Ai was 0.941 (*p* = 0.4705), with no statistical difference (Table 2; Figure 4A). To compare the optimal cutoff values of consciousness states for Ai and BIS, the Youden index method of determining the cutoff value on the receiver operating characteristic curve was adopted. The value range was 0–1, and the optimal cutoff for the index monitoring was defined as the maximum value of the index. The optimal cutoff values for anesthesia-induced unconsciousness of the Ai and BIS were 71.5 and 72.5, respectively, and those for the Youden index were 0.798 and 0.814, respectively (Table 2; Figure 5).

**Table 2:** Comparison of the accuracy and optimal cutoff values in judging the anesthesia-induced unconsciousness, LOC, and ROC between Ai and BIS.

States of consciousness	Index	AUC	SE	95%CI	Cutoff value	Yd
Anesthesia-induced	Ai	0.941	0.00401	0.933–0.948	71.5	0.789
Unconsciousness	BIS	0.943	0.00412	0.935–0.950	72.5	0.814
LOC	Ai	0.953	0.00437	0.944–0.960	79.5	0.882
	BIS	0.965	0.00424	0.957–0.971	79.5	0.855
ROC	Ai	0.936	0.00456	0.927–0.944	70.5	0.799
	BIS	0.934	0.00479	0.925–0.942	71.5	0.778

AUC, Area under the curve; SE, Standard error; CI: Confidence interval; Cutoff, Optimal Cutoff value; Yd, Youden index.

To compare the accuracy and optimal cutoff values of Ai and BIS in distinguishing LOC, the receiver operating characteristic curve analysis showed that the AUC of BIS in discriminating the LOC was 0.953, and the AUC of Ai was 0.965 (*p* = 0.0013), with a statistical difference (Table 2; Figure 4B). The optimal cutoff values for the LOC of the two indices were both 79.5, while those of the Youden index were 0.882 and 0.855, respectively (Table 2).

To compare the accuracy and optimal cutoff value of Ai and BIS in judging ROC, receiver operating characteristic curve analysis revealed that the AUC of BIS in discriminating the ROC was 0.936, and the AUC of Ai was 0.934 (*p* = 0.5271), with no statistical difference (Table 2; Figure 4C). The optimal cutoff values for the ROC of the two indices were 71.5 and 70.5, respectively, and those of the Youden index were 0.799 and 0.778, respectively (Table 2).

The receiver operating characteristic curve analysis was used to compare the accuracy and optimal cutoff values of Ai and BIS in distinguishing the anesthesia-induced unconsciousness, LOC, and ROC in patients below and over 60 years of age. The results showed that Ai and BIS discriminated the different states of consciousness similarly. However, the optimal cutoff values for patients aged 60 years and above were slightly lower than that of patients below 60 years old, but the difference was not significant (Table 3).

**Table 3:** Comparison of the efficacy of Ai and BIS in monitoring the different states of consciousness of patients in different age groups.

States of consciousness	Index	Age (years)	AUC	SE	95% CI	Cutoff value
Anesthesia-induced unconsciousness	Ai	<60	0.947	0.004779	0.852, 0.942	71.5
		≥60	0.932	0.006998	0.866, 0.938	70.5
	BIS	<60	0.947	0.005216	0.879, 0.937	72.5
		≥60	0.939	0.006647	0.892, 0.919	72.5
LOC	Ai	<60	0.958	0.005202	0.889, 0.967	78.5
		≥60	0.945	0.007534	0.882, 0.977	75.5
	BIS	<60	0.969	0.005027	0.929, 0.967	79.5
		≥60	0.960	0.007158	0.917, 0.953	77.5
ROC	Ai	<60	0.943	0.005514	0.852, 0.923	71.5
		≥60	0.926	0.007846	0.863, 0.922	69.5
	BIS	<60	0.937	0.006133	0.862, 0.942	70.5
		≥60	0.930	0.007623	0.869, 0.929	69.5

AUC, Area under the curve; SE, Standard error; CI, Confidence interval; Cutoff, Optimal cutoff value.

### Stability comparison of Ai and BIS during noxious stimulation

Spearman’s rank correlation analysis was used to compare the correlation between Ai and BIS and the hemodynamics during noxious stimulation. This analysis showed that there was no correlation between Ai and BIS with blood pressure (systolic arterial pressure and diastolic arterial pressure) and heart rate during noxious stimulation (tracheal intubation, surgical skin incision, establishment of pneumoperitoneum, peritoneal irrigation, and endoscope removal) under anesthesia (Table 4).

**Table 4:** Correlation analysis of Ai and BIS with hemodynamics during noxious stimulation.

Index	Hemodynamics (during noxious stimulation)	Correlation coefficient	*p* value
BIS	SAP	−0.004	0.906
	DAP	0.010	0.754
	HR	0.011	0.736
Ai	SAP	−0.019	0.556
	DAP	0.009	0.788
	HR	0.028	0.385

SAP, Systolic arterial pressure; DAP, Diastolic arterial pressure; HR, Heart rate.

## Discussion

Our study evaluated the consistency, accuracy, and stability of the Ai and BIS in monitoring the DOA during sevoflurane anesthesia under prospective multi-center randomized controlled conditions. The Ai (ConView YY-105), a new anesthesia depth monitor, differs from the BIS in terms of the underlying algorithm. BIS monitors the mixed information obtained by fast Fourier transform and bispectral analysis of the power and frequency of the EEG and classifies this information as an optimal number on a scale of 0–100, with zero indicating no EEG signal and 100 indicating awakeness. The changes in these values can reflect the functional status of the cerebral cortex (Vretzakis et al., 2005). Conversely, after anesthesia, Ai classified EEG signals using a decision tree classifier, and anesthesia awareness index was obtained through the least squares method (Liu et al., 2015). The design process of the Ai defines the appropriate anesthesia depth at 40–60, which is consistent with that of the BIS (Liu et al., 2015).

A study reported that under general anesthesia with propofol, Ai exhibits a good correlation with BIS, which is widely used in clinical practice (Fu et al., 2019). However, compared with propofol, inhalation anesthetics have different effects on EEG, including biphasic changes, which may be related to the increased activity of hormones, such as dopamine, in the body (Boisseau et al., 2002). As an ideal inhalation anesthetic, sevoflurane has a low blood gas partition coefficient, which makes the onset and recovery of anesthesia faster, allowing the DOA to be well controlled. The BIS correlates with the DOA produced by most anesthetics and is reliable for monitoring DOA with sevoflurane (Ibrahim et al., 2001). Therefore, in this study, we maintained a BIS of 40–60 as the appropriate DOA under sevoflurane while monitoring Ai synchronously in the same patient. This allowed us to observe the consistency between Ai and BIS.

Bland–Altman analysis (Bland and Altman, 1986; Bland and Altman, 2007) showed that the mean difference between Ai and BIS was not significant (−0.1747, 95% confidence interval (−0.6660 to 0.3166), *p* = 0.4857), indicating that Ai and BIS monitoring have high consistency and both can accurately reflect the DOA of patients. The results of the Bland–Altman consistency evaluation of Ai and BIS were similar to those reported by Fu et al. (2019).

Previous studies have identified Deming regression analysis as a valid method to analyze the agreement between clinical monitoring methods, and it can be used to assess whether there is a fixed or proportional bias between the two monitoring methods (Haji et al., 2015; Nasr et al., 2019). In this study, the Deming regression analysis was *y* = 5.6387 + 0.9067*x* (*y* is BIS, *s* is Ai), and the slope and intercept were both statistically significant. This revealed similar DOA values for Ai and BIS monitoring, with high consistency and no systematic deviation in the readings. We further demonstrated that both Ai and BIS can objectively monitor the DOA of patients under sevoflurane.

Receiver operating characteristic curve analysis is a commonly used and effective clinical judgment method. Researchers have used it to judge the success rate of weaning in elderly high-risk heart disease patients (Bouhemad et al., 2020) and to judge acute kidney injury after cardiac surgery (Coulson et al., 2021). Thus, the receiver operating characteristic curve analysis is also applicable in evaluating the accuracy of Ai and BIS for discriminating the DOA. In addition, the Youden index was used to determine the optimal cutoff value on the receiver operating characteristic curve, and the range of values was 0–ss1. The maximum index value was the optimal cutoff value for the monitoring method.

In this study, the receiver operating characteristic curve was used to compare the accuracy and optimal cutoff values of Ai and BIS in evaluating anesthesia-induced unconsciousness. The optimal cutoff values for Ai and BIS for anesthesia-induced unconsciousness were 71.50 and 72.50, respectively. Previous studies have reported that the difference between various anesthesia depth index values is significant if the difference is greater than or equal to 10 (Aho et al., 2015). Therefore, the difference in the optimal cutoff value of anesthesia-induced unconsciousness between Ai and BIS is not clinically significant. The results of this study show that the accuracy of Ai and BIS in judging the level of consciousness is consistent, and the optimal cutoff values of anesthesia-induced unconsciousness are similar.

The receiver operating characteristic curve was used to further analyze the accuracy and optimal cutoff values of Ai and BIS in distinguishing LOC and ROC. Our results showed that the AUC of BIS for distinguishing LOC was 0.953, and the AUC of Ai was 0.965 (*p* < 0.05), suggesting that the accuracy of BIS was slightly higher than that of Ai in judging the LOC in adults, although the difference in AUC was 0.012, which is very small. Statistically, when the sample size is large enough, a small difference may show a significant statistical difference, but the clinical significance of this difference will be small; therefore, it can be considered that the accuracies of Ai and BIS in distinguishing LOC are similar. However, there was no statistically significant difference in the AUC between Ai and BIS, and the accuracies were the same. In this study, Ai and BIS had the same optimal cutoff value for LOC (79.5), whereas the optimal cutoff value for ROC in Ai (71.5) was different from that of BIS (70.5), but the difference was not clinically significant.

A study reported that when propofol induced unconsciousness, the mean values of Ai and BIS were 60.76 and 62.18, respectively, at LOC and 73.9 and 75.66, respectively, at ROC (Bouhemad et al., 2020). Compared with the results of this study, the mean value of LOC during anesthesia induction with Ai and BIS was lower than our optimal cutoff value, which may be related to the inconsistent interval of consciousness judgment during anesthesia induction. In this study, during induction, patients were instructed to open their eyes, and verbal communication with the patient continued while the anesthetists assessed their state of consciousness.In previous studies, the time interval for assessing the level of consciousness during anesthesia induction was 30 s (Bouhemad et al., 2020). A possible reason is that it takes a certain amount of time to obtain an Ai or BIS through the acquisition of EEG signals and proprietary algorithms, resulting in a significant time delay for Ai or BIS to display visible values (Li and Li, 2014).

It was reported that under the same concentration of sevoflurane, the decreased magnitude of BIS of patients in the elderly group (age > 60 years) is greater than that in the young group (age 20–40 years), which may be related to the decline in nervous system function and increase in sensitivity to anesthetic drugs in elderly patients (Katoh et al., 1993). Receiver operating characteristic curve analysis was used to identify any differences in the accuracy and optimal cutoff values of Ai and BIS for different age groups. We found that Ai and BIS had similar accuracies and optimal critical values in discriminating anesthesia-induced unconsciousness, LOC, and ROC in young and middle-aged or elderly patients, and there was no statistical difference. Therefore, the results of this study show that the accuracy and optimal cutoff values of the two indices for distinguishing anesthesia-induced unconsciousness, LOC, and ROC are insensitive to age.

Many studies have investigated the effect of Ai in monitoring the DOA in patients under non-noxious stimulation (Liu et al., 2015; Fu et al., 2019). However, the influence of hemodynamic fluctuations on Ai and BIS during intraoperative noxious stimulation remains unclear. In this study, surgeries, such as tracheal intubation, surgical skin incision, establishment of pneumoperitoneum, peritoneal irrigation, and endoscope removal under anesthesia, were considered strong external noxious stimuli (Chen et al., 2010). We aimed to evaluate the stability of DOA monitoring by comparing the correlation between Ai and BIS with hemodynamics during noxious stimulation. The results revealed that Ai and BIS were not correlated with blood pressure and heart rate during noxious stimulation, indicating that the stability of Ai monitoring of anesthesia depth was similar to that of BIS and was not affected by hemodynamic fluctuations.

Our study has several limitations. First, the anti-jamming capabilities of Ai and BIS were not evaluated. The duration of intraoperative interference, such as electrosurgical interference, is relatively short, and real-time observation and recording are required for comparison. However, we mainly compared the changes in the level of consciousness of Ai and BIS during anesthesia, especially during anesthesia induction and awakening. No obvious abnormal interference occurred because of the short observation time. Furthermore, relevant interference times and events were not recorded. Previous studies have reported that opioid analgesics have no effect on BIS (Lysakowski et al., 2001). Therefore, this study did not consider the effect of opioid analgesics on the DOA index. Moreover, muscle relaxants reportedly have no direct effect on EEG signals, but can suppress electrical activity in the frontal muscles, which may interfere with the accuracy of the DOA index. To avoid the possible influence of muscle relaxants on the DOA index, cisatracurium was administered only after LOC was assessed during anesthesia induction, and additional muscle relaxants were stopped 30 min before the end of the surgery to minimize the effect of muscle relaxants on anesthesia. Minimize the effect of muscle relaxants on EEG signal acquisition and analysis. In this study, the effect of noxious stimulation intensity on the anesthesia depth index was evaluated only by monitoring changes in blood pressure and heart rate. The measurement of catecholamines in the blood of patients should be assessed to further reflect patient intraoperative stress, to provide more accurate evidence for changes in the DOA index.

In our study, Ai and BIS showed high consistency, similar accuracies, and good stability in DOA monitoring of sevoflurane anesthesia in 10 medical centers. In addition, Ai-based DOA monitoring results are insensitive to age and not affected by hemodynamic fluctuations during intraoperative noxious stimulation which provide broader prospects for clinical research.

## Data availability statement

The original contributions presented in the study are included in the article/supplementary material, further inquiries can be directed to the corresponding authors.

## Ethics statement

The studies involving human participants were reviewed and approved by the Medical Ethics Committee of the Second Affiliated Hospital of Army Medical University (NO. 2020-Research No. 062–01). The patients/participants provided their written informed consent to participate in this study.

## Author contributions

LH, JZhan and QD: conceived and designed the experiments. FC, ZW, ZD, JZeng, LH, JZhang, YS, KL, and MW: performed the experiments. JZhan, ZD, FC and ZW: collected data. QD, JZhan, and FC: analyzed data. HL, JZhan, FC, QD and ZW: wrote the paper. All authors contributed to the article and approved the submitted version.

## Funding

This study is supported by the National Key R&D Program of China (2018YFC0117200) and the Clinical Research Project of Army Medical University (CX2019LC114).

## Conflict of interest

The authors declare that the research was conducted in the absence of any commercial or financial relationships that could be construed as a potential conflict of interest.

The handling editor YW declared a shared parent affiliation with the author JZ at the time of review.

## Publisher’s note

All claims expressed in this article are solely those of the authors and do not necessarily represent those of their affiliated organizations, or those of the publisher, the editors and the reviewers. Any product that may be evaluated in this article, or claim that may be made by its manufacturer, is not guaranteed or endorsed by the publisher.
